# Atrial fibrillation prediction by combining ECG markers and CMR radiomics

**DOI:** 10.1038/s41598-022-21663-w

**Published:** 2022-11-07

**Authors:** Esmeralda Ruiz Pujadas, Zahra Raisi-Estabragh, Liliana Szabo, Cristian Izquierdo Morcillo, Víctor M. Campello, Carlos Martin-Isla, Hajnalka Vago, Bela Merkely, Nicholas C. Harvey, Steffen E. Petersen, Karim Lekadir

**Affiliations:** 1 Artificial Intelligence in Medicine Lab (BCN-AIM), Departament de Matemàtiques i Informàtica, Universitat de Barcelona, Barcelona, Spain; 2 William Harvey Research Institute, NIHR Barts Biomedical Research Centre, Queen Mary University of London, Charterhouse Square, London, EC1M 6BQ UK; 3 Barts Heart Centre, St Bartholomew’s Hospital, Barts Health NHS Trust, West Smithfield, London, EC1A 7BE UK; 4 Semmelweis University Heart and Vascular Center, Budapest, Hungary; 5 MRC Lifecourse Epidemiology Centre, University of Southampton, Southampton, UK; 6 NIHR Southampton Biomedical Research Centre, University of Southampton, University Hospital Southampton NHS Foundation Trust, Southampton, UK; 7 Health Data Research UK, London, UK; 8 Alan Turing Institute, London, UK

**Keywords:** Electrodiagnosis, Cardiovascular diseases, Arrhythmias, Heart failure, Machine learning

## Abstract

Atrial fibrillation (AF) is the most common cardiac arrhythmia. It is associated with a higher risk of important adverse health outcomes such as stroke and death. AF is linked to distinct electro-anatomic alterations. The main tool for AF diagnosis is the Electrocardiogram (ECG). However, an ECG recorded at a single time point may not detect individuals with paroxysmal AF. In this study, we developed machine learning models for discrimination of prevalent AF using a combination of image-derived radiomics phenotypes and ECG features. Thus, we characterize the phenotypes of prevalent AF in terms of ECG and imaging alterations. Moreover, we explore sex-differential remodelling by building sex-specific models. Our integrative model including radiomics and ECG together resulted in a better performance than ECG alone, particularly in women. ECG had a lower performance in women than men (AUC: 0.77 vs 0.88, p < 0.05) but adding radiomics features, the accuracy of the model was able to improve significantly. The sensitivity also increased considerably in women by adding the radiomics (0.68 vs 0.79, p < 0.05) having a higher detection of AF events. Our findings provide novel insights into AF-related electro-anatomic remodelling and its variations by sex. The integrative radiomics-ECG model also presents a potential novel approach for earlier detection of AF.

## Introduction

Atrial fibrillation (AF) is the most common cardiac arrhythmia. It is characterized by an irregular heart rhythm and often abnormally rapid heart rate. The most common complications of AF are increased risk of stroke, heart failure, and death^1^. These complications may be mitigated by early AF detection and initiation of appropriate treatments, such as anticoagulation and rate control therapies.

Cardiac structure and electrical activity are two important, inter-linked aspects of cardiac health and disease. The cardiac conduction system is complex and depends on the global and local structure of the cardiac chambers. The occurrence of AF is linked to distinct electro-anatomic cardiovascular remodeling^2^.

Electrical recordings of the heart such as the 12-lead electrocardiograms (ECG) provide indications of cardiovascular health. The ECG is a dynamic physiological signal that represents the electrical activity of the heart. It is widely used to identify patterns or abnormalities in cardiac rhythms and waveforms. ECG recordings are the main clinical tool for AF diagnosis^3^. The best indicators are the absence of the p-wave degenerating into small magnitude fibrillatory waves and the irregularity of R–R intervals indicating irregular conduction of atrial impulses through the atrioventricular (AV) node to the ventricles. The study of the QRS complex, a combination of the Q wave, R wave and S wave that represents ventricular depolarization, might also add some information by analyzing the height of the amplitude or the size of the interval. But the latter indicator might have normal values even when the AF is present^4^. Furthermore, AF frequently occurs intermittently with the characteristic AF-defining features only apparent when an individual experiences a paroxysm of AF. Whilst paroxysmal AF is more challenging to diagnose, it confers the same adverse risks as individuals continuously in an AF rhythm^5^.

Emerging deep learning approaches have shown promise in quantifying complex patterns in cardiac electrical activity^6,7^. However, there is room for improvement. For patients with undiagnosed AF, ischemic stroke may be the first clinical manifestation of the condition. AF is detected for first time in approximately one-fourth of patients presenting with ischemic stroke^8,9^. Early detection of AF may enable early intervention and prevention of ischemic stroke.

There are numerous conference challenges, particularly organized by Physionet, which aim to address early detection using machine learning techniques^10^. In spite of the successful results, the existing works in the literature do not stratify by sex. This is an important consideration given significant sex differential patterns in AF highlighted in clinical papers. The estimated prevalence of AF is lower in women, whilst this may reflect genuine lower burden of AF in women it may also indicate under-diagnosis in this population^11^. Indeed, women with AF experience higher mortality and ischemic stroke and are less often prescribed anticoagulation treatments^12^.

Cardiovascular Magnetic Resonance Imaging (CMR) plays an important role in the diagnosis of complex cardiac diseases. Recently, the concept of radiomics has attracted significant attention in the cardiac imaging community^13^ due to its ability to quantify and analyse large pools of advanced imaging phenotypes, which are descriptive of complex shape, size, intensity or textural patterns. Preliminary results have shown the promise of CMR radiomics for AF discrimination^14^.

CMR radiomics extracts a large number of quantitative features using data characterization algorithms. These techniques are very promising for deeper image phenotyping of cardiac structure and tissue^15^.

The combination of imaging phenotypes and ECG features for AF detection has not been explored in the existing literature. Yet, such an approach may enable integration of complementary signals and hence improve AF detection by considering both anatomical and electrical alterations.

In this work, we aim to evaluate the feasibility of combining cardiac imaging with ECG features for AF detection considering sex-differential patterns. Integrated risk prediction models were built combining CMR radiomics and ECG parameters, separately for men and women. Morphological, temporal and non-linear features were extracted from the ECG waveforms. The study was performed using the UK Biobank resource, a large-scale health database publicly available under request. To our knowledge, it is the first time, that the combination of ECG and imaging are explored. The inclusion of radiomics allows a more precise information of the AF event and quantifies the complexity of cardiac structure and remodeling providing a complementary information additionally to the ECG test.

## Related work

The related works can be divided into three categories according to the computation of the features: ECG features extracted from the waveforms with machine learning techniques, ECG features extracted from deep learning methods and hybrid frameworks that combines traditional ECG features with the ones extracted using deep learning algorithms.

Classical approaches were mainly based on morphological features of the ECG signal in time domain such as heartbeat, analysis of intervals and amplitudes of QRS, QT, PR and R–R^16,17^. Those studies, with satisfactory results, may be sensitive to the ECG noise. To alleviate this issue, the morphological features were computed in other domains such as in the frequency or time–frequency domain. Some examples of these features are power spectral density of the R-R intervals and frequency bands (e.g., ultra low , very low and low). Non-linear features were also considered as the model of the heart cannot be reduced to a linear function as it also involves a nonlinear contribution^18,19^. Some works combining ECG features in different domains are the following: Yin et al. proposed a multi-domain ECG feature extraction method^20^. The multi-domain features were composed of nonlinear and frequency domain features, which were used as input features to train and test an SVM classifier model. Zabihi et al. also proposed a multi-domain ECG feature extraction which included time-domain features, time–frequency, phase-space based on non-linear features and meta-level information^21^. Random forest classifier was applied for feature selection as well as for classification.

As the technology evolves, more data can be processed, and deep learning techniques emerge. Many works have applied deep learning for feature extraction using convolutional neural networks (CNN)^22–24^, long and short memory networks (LSTM)^25,26^ as well as their variants^27–29^.

Other studies have applied deep learning to obtain new features and fused them with traditional features. Some examples are the following. Smoleń created an initial model using Recurrent Neural Network (RNN) classifier, that was fed by lengths of intervals between following R peaks. The computed probabilities for each class were combined with hand-designed features and used as an input for Gradient Boosting Machine (GBM) classifier^30^. The features selected were categorized into 5 categories: statistical features, QRS morphology features, RR-interval features, noise features, and frequency-based features. The performance of those methods was very promising. Most of them used publicly available databases within conference challenges. However, the main issue of deep learning techniques is that a large number of samples are required in order to ‘learn’ and generally, hospitals do not have enough cases to use this type of methods.

Additionally, the AF might not be registered on standard of care 12-lead ECG during hospital visits, opening a new line of research into AF detection using portable devices such as smartwatches. Those portable devices might not be as sophisticated and exact as clinical ECG devices. But it has the advantage that it can deal with the early detection of the AF as long periods of signal are recorded.

Screening is suggested as one strategy to increase AF detection rates and start anticoagulation in an earlier stage in high-risk individuals. Screening by opportunistic pulse palpation or ECG rhythm strip is already recommended by the European Society of Cardiology (ESC) in all patients older than 65 years contacting health services and by the National Institute for Health and Care Excellence (NICE) where patients have a symptom suggestive of AF^29^.

Another line of research is using risk factors, biomarkers, ECGs or a combination of risk factors and imaging features in order to predict incident cases of AF. A strong causal relationship between natriuretic peptides NT-proBNP, BNP and MR-proANP, and incidence of AF was ruled out by Geelhoed et al.^31^. Vascular risk factors including diabetes, hypertension as well as daily lifestyle variables such as smoking and obesity^32,33^ have also been studied in relation to incident atrial fibrillation as well as the inclusion of CMR Imaging^34^, in recent studies. ECG features have also been analyzed to study the possibility to develop AF^35^. In spite of the promising results, the studies are in an initial stage of research and have not been integrated in clinical routine.

## Results

### Baseline characteristics

We studied 32,121 UK Biobank participants with an average age of 63 (± 7.53) years. 51% of the participants were female. A total of 495 participants had prevalent AF. The AF cohort included a greater proportion of men (69.3%), slightly older individuals with greater comorbidity burden, and higher BMI. For most baseline metrics there was no statistically significant difference between men and women except in education level and alcohol intake. Specifically, men were more likely to participate in higher education (48% vs. 34%) than women and consume alcohol more than 1–2 time a week. The Table 1 summarizes the baseline characteristics.

**Table 1:** Baseline characteristics describing the whole population, population without AF, patients with AF in general and sex-specific.

Characteristics	Whole population (n = 32,121)	Subjects without AF (n = 31,424)	Patients with AF (n = 495)	p-value AF vs non-AF	AF in women (n = 152)	AF in men (n = 343)	p-value AF in women vs men
Age mean (std)	63.27 (± 7*.*53)	63.16 (± 7.52)	68 (± 6.43)	< 0.001	68.04 (± 6.54)	68.61 (± 6.55)	**0.29**
Female sex n (%)	16,658 (51.86%)	16,442 (52.32%)	152 (30.70%)	< 0.001	152 (100%)	0 (0%)	
Townsend deprivation index median (IQR)	−1.95 (3.30)	−2.64 (3.30)	−2.74 (3.54)	**0.66**	−2.73 (3.50)	−2.74 (3.55)	**0.43**
Body mass index mean (kg/m^2^)	26.57 (± 4*.*35)	26.55 (± 4*.*34)	27.79 (± 4.53)	< 0.001	27.91 (± 5.41)	27.74 (± 4.09)	**0.73**
Current smoker n (%)	2032 (6.32%)	1993 (6.34%)	26 (5.25%)	**0.32**	5 (3.28%)	21 (6.12%)	**0.19**
Diabetes status n (%)	993 (3.09%)	963 (3.06%)	20 (4.04%)	**0.21**	6 (3.94%)	14 (4.08%)	**0.94**
Hypertension status n (%)	4397 (13.68%)	4177 (13.29%)	165 (33.33%)	< 0.001	48 (31.57%)	117 (34.11%)	**0.58**
High cholesterol status n (%)	7272 (22.63%)	7055 (22.45%)	164 (33.13%)	< 0.001	42 (27.63%)	122 (35.56%)	**0.08**
IPAQ (MET minutes/week) median [IQR]	2271 [2360]	1528 [2350]	1532 [2545]	**0.84**	1543 [2772]	1515 [2373]	**0.99**
**Education level n (%)**				**0.56**			0.002
Left school age 14 or younger	421 (1.31%)	414 (1.31%)	5 (1.01%)		2 (1.31%)	3 (0.87%)	
Left school age 15 or older	2260 (7.03%)	2198 (6.99%)	46 (9.29%)		18 (11.84%)	28 (8.16%)	
High school diploma	4229 (13.16%)	4138 (13.16%)	59 (11.91%)		27 (17.76%)	32 (9.32%)	
Sixth form qualification	1820 (5.66%)	1785 (5.68%)	24 (4.84%)		5 (3.28%)	19 (5.53%)	
Professional qualification	8953 (27.87%)	8745 (27.82%)	143 (28.88%)		48 (31.57%)	95 (27.69%)	
Higher education University degree	14,438 (44.94%)	14,144 (45.01%)	218 (44.04%)		52 (34.21%)	166 (48.39%)	
**Alcohol intake n (%)**	16,658 (51.86%)			< 0.001			< 0.001
Never	−1.95 (3.30)	1505 (4.78%)	28 (5.65%)		13 (8.55%)	15 (4.37%)	
Special occasions only	26.57 (± 4*.*35)	2601 (8.27%)	35 (7.07%)		20 (13.15%)	15 (4.37%)	
1–3 times a month	2032 (6.32%)	3393 (10.79%)	40 (8.08%)		21 (13.81%)	19 (5.53%)	
1–2 times a week	993 (3.09%)	8133 (25.88%)	109 (22.02%)		31 (20.39%)	78 (22.74%)	
3–4 times a week	4397 (13.68%)	8896 (28.30%)	133 (26.86%)		36 (23.68%)	97 (28.27%)	
Daily or almost daily	7272 (22.63%)	6896 (21.94%)	50 (26.31%)		31 (20.39%)	119 (34.69%)	

In bold, the characteristics with not significant differences.

### Correlation between ECG and Radiomics features

**Figure 1:**
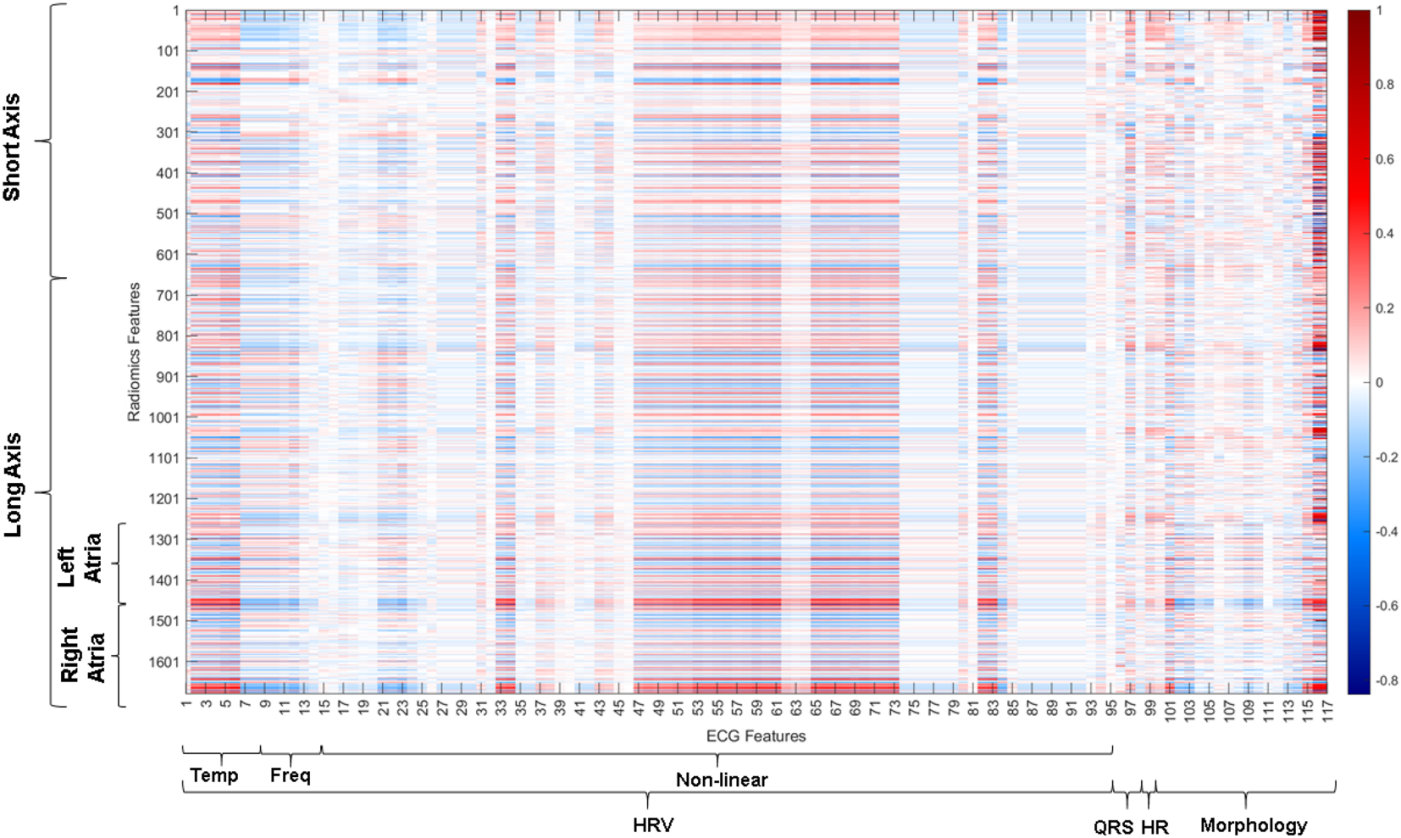
Correlation between ECG and radiomics features showing low correlation between radiomics features extracted from the short-axis images and a slightly higher correlation with the features from the long axis images including atrial metrics. *Temp* temporal, *Freq* frequency, *HRV* heart rate variability, *HR* heart rate.

The correlation between ECG and radiomics features was not very high, as illustrated in Fig. 1. The morphological features were the ones with a certain correlation along all the radiomics features both in short and in long axis. Moreover, a higher correlation is shown for radiomics features computed from long axis images (vs short axis) as these features include atrial radiomics, particularly in the heart rate variability in temporal and non-linear domain. Thus, ECG features seem to have a higher correlation with the features related to the atria than with the other regions of interest of the heart. However, the correlation found is not high between ECG and radiomics with the two providing additive and complementary information.

### Feature selection for each model

For the model that includes both sexes, the ECG features which are related to the heart rate (such as tachycardia) were the most predominant features. The volume and surface of the left atrial were also important features in the model. Most of the relevant radiomics features are first, shape and then texture. The region of interest (ROI) selected for all the features are left atrium (LA) and the phase end diastole (ED). In Table 2, all the selected features are shown for the general model.

**Table 2:** Feature selection for AF for all participants for the electro-radiomics model.

Feature selection	Feature type	ROI	Phase	Importance
'tachy'				95.7189000000000
'Clvl6'				95.3946000000000
'brady'				94.4181000000000
'LA_shape_SurfaceArea_ED'	Shape	LA	ED	93.3183000000000
'LA_shape_VoxelVolume_ED'	Shape	LA	ED	93.0671000000000
'LA_shape_MeshVolume_ED'	Shape	LA	ED	92.9818000000000
'medianRR'				92.2226000000000
'LA_shape_Sphericity_ED'	Shape	LA	ED	91.1313000000000
'meanRR'				89.5061000000000
'LA_shape_SurfaceVolumeRatio_ED'	Shape	LA	ED	86.7621000000000
'DistCennS'				86.5382000000000
'LA_gldm_DependenceNonUniformity_ED'	Texture	LA	ED	86.0905000000000
'LA_shape_Maximum2DDiameterColumn_ED'	Shape	LA	ED	85.0250000000000
'LA_shape_Maximum2DDiameterSlice_ED'	Shape	LA	ED	84.4272000000000
'LA_shape_Maximum3DDiameter_ED'	Shape	LA	ED	84.4272000000000
'LA_firstorder_Energy_ED'	First-Order	LA	ED	84.0768000000000
'LA_firstorder_TotalEnergy_ED'	First-Order	LA	ED	83.5177000000000
'LA_shape_MajorAxisLength_ED'	Shape	LA	ED	83.3500000000000
'LA_ngtdm_Strength_ED'	Texture	LA	ED	77.9337000000000
'LA_shape_MinorAxisLength_ED'	Shape	LA	ED	77.4875000000000
'LA_glrlm_GrayLevelNonUniformity_ED'	Texture	LA	ED	75.8153000000000
'LA_glszm_GrayLevelNonUniformity_ED'	Texture	LA	ED	74.2248000000000
'LA_ngtdm_Busyness_ED'	Texture	LA	ED	74.0887000000000
'Dlvl2'				73.8246000000000
'Dlvl8'				73.8246000000000
'LA_glrlm_RunLengthNonUniformity_ED'	Texture	LA	ED	72.1410000000000
'LA_ngtdm_Coarseness_ED'	Texture	LA	ED	69.0288000000000
'LA_gldm_GrayLevelNonUniformity_ED'	Texture	LA	ED	67.3082000000000
'Dlvl3'				65.5733000000000
'Dlvl9'				65.5733000000000

For the separate model in women, the most predominant features are mean of RR and diameter of the LA. The shape and texture variables are also informative model features. The ranking of importance is lower than the other models. In the model built with only male participants, the sphericity and volume of the LA are selected as the most relevant features followed by the ECG features such as tachycardia and bradycardia. The shape features are the most selected and secondly the texture.

In Tables 3 and 4, the features for the female and the male are described with the ranking score. In Table 5, the repeatability of the variables in women is also shown in the partitions of the nested-cross validation.

**Table 3:** Feature selection for AF for women for the electro-radiomics model.

Feature selection	Feature type	ROI	Phase	Importance
'meanRR'				23.8432000000000
'LA_shape_Maximum2DDiameterColumn_ED'	Shape	LA	ED	23.2997000000000
'Clvl6'				23.2829000000000
'brady'				22.6090000000000
'LA_shape_Sphericity_ED'	Shape	LA	ED	22.1852000000000
'medianRR'				21.1193000000000
'tachy'				20.5118000000000
'LA_shape_MinorAxisLength_ED'	Shape	LA	ED	20.4190000000000
'LA_firstorder_TotalEnergy_ED'	First-Order	LA	ED	20.3422000000000
'LA_shape_VoxelVolume_ED'	Shape	LA	ED	20.2966000000000
'LA_shape_MeshVolume_ED'	Shape	LA	ED	20.2541000000000
'LA_shape_SurfaceArea_ED'	Shape	LA	ED	20.2347000000000
'Dlvl2'				20.1606000000000
'Dlvl8'				20.1606000000000
'LA_shape_SurfaceVolumeRatio_ED'	Shape	LA	ED	20.0505000000000
'LA_firstorder_Energy_ED'	First-Order	LA	ED	19.5947000000000
'LA_gldm_DependenceNonUniformity_ED'	Texture	LA	ED	19.3729000000000
'LA_glrlm_RunLengthNonUniformity_ED'	Texture	LA	ED	19.2391000000000
'LA_shape_MajorAxisLength_ED'	Shape	LA	ED	19.0635000000000
'LA_glrlm_GrayLevelNonUniformity_ED'	Texture	LA	ED	18.6670000000000
'DistCennS'				18.3854000000000
'RA_shape_MajorAxisLength_ED'	Shape	RA	ED	17.3390000000000
'LA_shape_Maximum2DDiameterColumn_ES'	Shape	LA	ES	17.3050000000000
'Dlvl3'				17.0507000000000
'Dlvl9'				17.0507000000000
'LA_glszm_GrayLevelNonUniformity_ED'	Texture	LA	ED	16.8258000000000
'LA_ngtdm_Coarseness_ED'	Texture	LA	ED	16.6224000000000
'LA_ngtdm_Strength_ED'	Texture	LA	ED	16.3367000000000
'RA_shape_Maximum2DDiameterSlice_ED'	Shape	RA	ED	15.9814000000000
'RA_shape_Maximum3DDiameter_ED'	Shape	RA	ED	15.9814000000000

**Table 4:** Feature selection for AF for men for the electro-radiomics model.

Feature selection	Feature type	ROI	Phase	Importance
'LA_shape_Sphericity_ED'	Shape	LA	ED	67.1634000000000
'LA_shape_VoxelVolume_ED'	Shape	LA	ED	66.8851000000000
'LA_shape_MeshVolume_ED'	Shape	LA	ED	66.6944000000000
'LA_shape_SurfaceArea_ED'	Shape	LA	ED	65.4330000000000
'LA_shape_Maximum2DDiameterSlice_ED'	Shape	LA	ED	64.5759000000000
'LA_shape_Maximum3DDiameter_ED'	Shape	LA	ED	64.5759000000000
'tachy'				64.0718000000000
'brady'				63.9600000000000
'LA_shape_MajorAxisLength_ED'	Shape	LA	ED	63.9363000000000
'LA_gldm_DependenceNonUniformity_ED'	Texture	LA	ED	63.8068000000000
'Clvl6'				63.3610000000000
'medianRR'				63.3370000000000
'LA_firstorder_TotalEnergy_ED'	First-Order	LA	ED	61.8981000000000
'LA_shape_SurfaceVolumeRatio_ED'	Shape	LA	ED	61.3106000000000
'LA_firstorder_Energy_ED'	First-Order	LA	ED	60.8644000000000
'DistCennS'				60.3365000000000
'meanRR'				59.1486000000000
'LA_shape_Maximum2DDiameterColumn_ED'	Shape	LA	ED	58.8432000000000
'LA_glrlm_GrayLevelNonUniformity_ED'	Texture	LA	ED	56.8149000000000
'LA_ngtdm_Busyness_ED'	Texture	LA	ED	53.9070000000000
'LA_ngtdm_Coarseness_ED'	Texture	LA	ED	53.7045000000000
'LA_shape_MinorAxisLength_ED'	Shape	LA	ED	53.6843000000000
'LA_glrlm_RunLengthNonUniformity_ED'	Texture	LA	ED	52.9183000000000
'LA_gldm_GrayLevelNonUniformity_ED'	Texture	LA	ED	51.8710000000000
'RA_shape_Sphericity_ED'	Shape	RA	ED	50.8045000000000
'LA_ngtdm_Strength_ED'	Texture	LA	ED	50.1410000000000
'RA_shape_MajorAxisLength_ED'	Shape	RA	ED	49.2244000000000
'LA_glszm_GrayLevelNonUniformity_ED'	Texture	LA	ED	49.1139000000000
'pNN50'				48.8595000000000
'RA_shape_VoxelVolume_ED'	Shape	RA	ED	48.5482000000000

**Table 5:** Feature selection for AF for women for the electro-radiomics model in all partitions in the nested-cross validation indicating the number of repetitions in each feature in each different partition.

Feature selection for AF in women in electro-radiomics model in all nested-cross validation partitions	Feature type	ROI	Phase	Number of iterations
‘meanRR'				10
‘LA_shape_Maximum2DDiameterColumn_ED'	Shape	LA	ED	10
‘Clvl6'				10
'brady'				10
'LA_shape_Sphericity_ED'	Shape	LA	ED	10
'medianRR'				10
'tachy'				10
'LA_shape_MinorAxisLength_ED'	Shape	LA	ED	8
'LA_firstorder_TotalEnergy_ED'	First-Order	LA	ED	10
'LA_shape_VoxelVolume_ED'	Shape	LA	ED	10
'LA_shape_MeshVolume_ED'	Shape	LA	ED	10
'LA_shape_SurfaceArea_ED'	Shape	LA	ED	10
'Dlvl2'				10
'Dlvl8'				9
'LA_shape_SurfaceVolumeRatio_ED'	Shape	LA	ED	10
'LA_firstorder_Energy_ED'	First-Order	LA	ED	10
'LA_gldm_DependenceNonUniformity_ED'	Texture	LA	ED	10
'LA_glrlm_RunLengthNonUniformity_ED'	Texture	LA	ED	10
'LA_shape_MajorAxisLength_ED'	Shape	LA	ED	10
'LA_glrlm_GrayLevelNonUniformity_ED'	Texture	LA	ED	8
'DistCennS'				10
'RA_shape_MajorAxisLength_ED'	Shape	RA	ED	5
'LA_shape_Maximum2DDiameterColumn_ES'	Shape	LA	ES	7
'Dlvl3'				9
'Dlvl9'				9
'LA_glszm_GrayLevelNonUniformity_ED'	Texture	LA	ED	5
'LA_ngtdm_Coarseness_ED'	Texture	LA	ED	7
'LA_ngtdm_Strength_ED'	Texture	LA	ED	7
'RA_shape_Maximum2DDiameterSlice_ED'	Shape	RA	ED	8
'RA_shape_Maximum3DDiameter_ED'	Shape	RA	ED	8
'LA_shape_Maximum2DDiameterSlice_ED'	Shape	LA	ED	9
'LA_shape_Maximum3DDiameter_ED'	Shape	LA	ED	8
'LA_ngtdm_Busyness_ED'	Texture	LA	ED	2
'LA_shape_MajorAxisLength_ES'	Shape	LA	ES	4
'RA_shape_Maximum2DDiameterColumn_ED'	Shape	RA	ED	2
'LA_shape_Maximum2DDiameterSlice_ES'	Shape	LA	ES	2
'edgebins2nL'				1
'LA_shape_Sphericity_ES'	Shape	LA	ES	1
'LA_shape_Maximum3DDiameter_ES'	Shape	LA	ES	1

**Figure 2:**
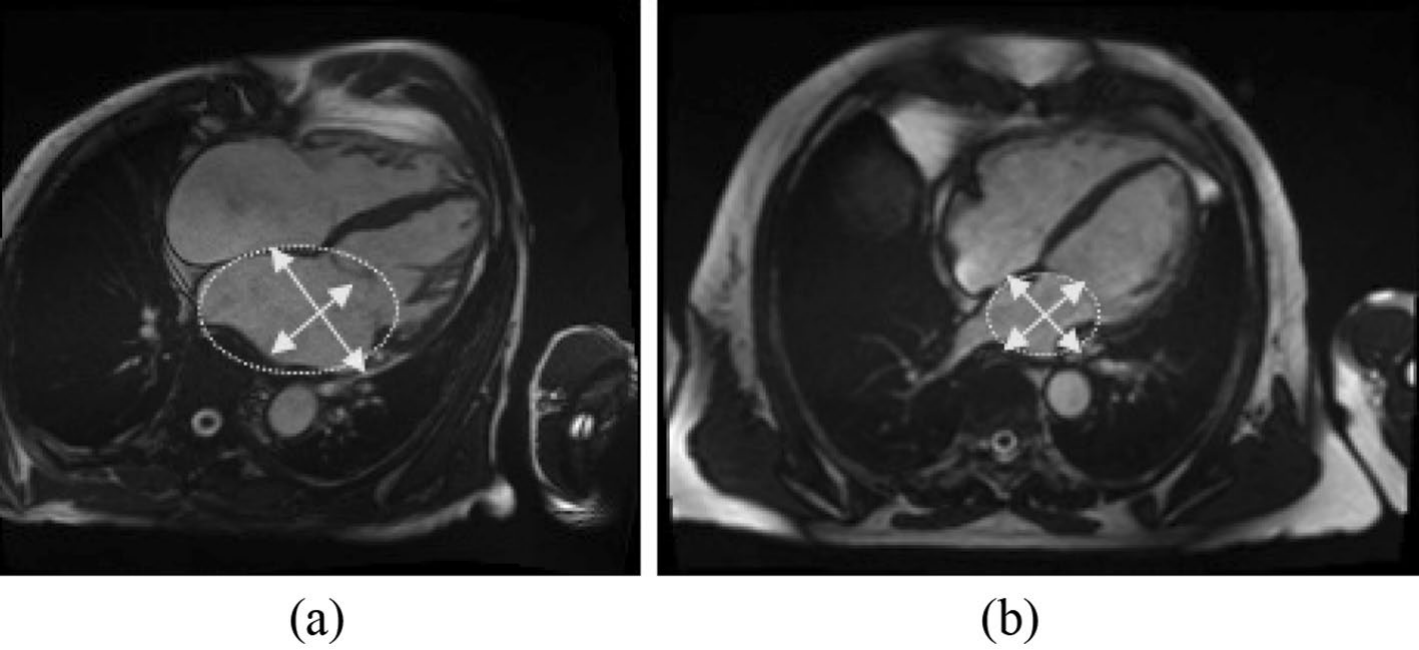
The figure shows four-chamber cine CMR images in end-diastole from two female UK Biobank participants. Our models selected the most important radiomics features from the left atrial region of interest. The arrows show the axis, and the circular shape indicates the sphericity. The first image (**a**) shows an AF patient with a larger axis and pronounced oval sphericity. The second image (**b**) illustrates a healthy subject with normal atrial dimensions, with more circular sphericity and a smaller axis than an AF patient.

In the three models, ED phase is selected the most and most of the radiomics features are from the left atrial ROI. The most predominant features are mainly based on shape and secondly textural features. First-order features do not have a high presence in the models. Figure 2 highlights visually the most relevant CMR markers, in the ED phase from the left atrial, for the women case but for men, it would be equivalent. The arrows indicate the axis, and the circular shape shows the sphericity. The AF patient has larger axis with a more oval sphericity than the healthy patient with a more circular shape of the left atrial.

### Performance of electro-radiomics models

**Figure 3:**
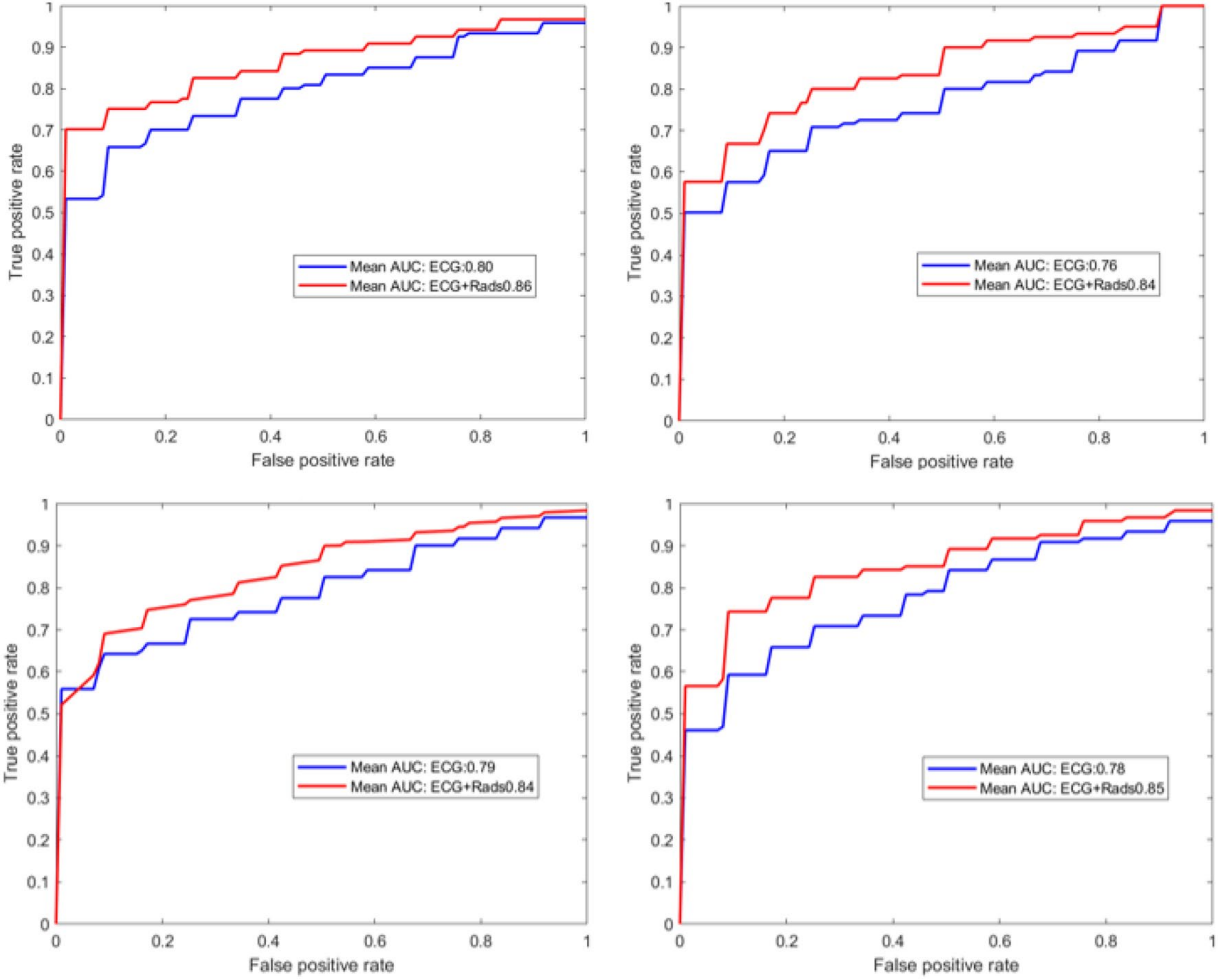
Different random partitions of the healthy cohort were randomly selected to show the added value of radiomics versus ECG alone in women. For all the cases, the improvement is clear and statistically significant (p < 0.05).

Table 6 shows the performance of the models adjusted by sex for the whole sample and for men and women separately. In the model with both sexes, radiomics did not show an added value compared with ECG alone or with the combination of both. In sex-specific analyses, we found poorer performance of the ECG model in women than men (AUC: 0.77 vs 0.88, p < 0.05). The addition of radiomics features improved the model accuracy for women to similar levels as for the ECG only model in men (AUC: 0.87 vs 0.88, p > 0.05). The sensitivity also increases considerably in women by adding the radiomics (0.68 vs 0.79, p < 0.05) having a higher detection of AF cases. According to our experiments, the addition of radiomics features has greater incremental value for AF discrimination in women than for men, where the added value is not clear. This behavior is not observed if we do not separate the data between men and women. To show, that the added value of radiomics in women does not depend on the data selected, we repeated the experiments with another randomly selected healthy comparator, observing consistent results throughout (Fig. 3). In order to test the robustness of the results with respect to covariates, we repeated the sex-specific experiments adjusting the models by: (i) age and sex (p > 0.05) and (ii) age, sex and main comorbidities related to AF which are diabetes, high cholesterol and hypertension (p > 0.05). The results in Table 7 follow the same pattern than the model adjusted by sex. It shows the robustness and strength of the features selected related to AF of the models.

**Table 6:** Average performance of the models for all AF patients adjusted by sex.

		ECG	Radiomics	ECG + radiomics
All	F1_score	0.82	0.71	0.81
	Accuracy	0.84	0.74	0.81
	Sensitivity	0.77	0.66	0.77
	Specificity	0.91	0.82	0.86
	AUC	0.86 (± 0.04)	0.82 (± 0.03)	0.87 (± 0.04)
Women	F1_score	0.72	0.72	0.78
	Accuracy	0.74	0.73	0.78
	Sensitivity	0.68	0.69	0.79
	Specificity	0.8	0.77	0.77
	AUC	0.77 (± 0.13)	0.81 (± 0.09)	0.87 (± 0.05)
Men	F1_score	0.84	0.73	0.82
	Accuracy	0.85	0.75	0.82
	Sensitivity	0.81	0.69	0.82
	Specificity	0.89	0.8	0.82
	AUC	0.88 (± 0.05)	0.82 (± 0.04)	0.89 (± 0.06)

The standard deviation is indicated in parenthesis.

**Table 7:** Average performance of the models for all AF patients adjusted by sex and age only, and sex age and other comorbidities.

		Adjusted by sex and age (p > 0.05)				Adjusted by sex and comorbidities (p > 0.05)		
			ECG	Radiomics	ECG + radiomics	ECG	Radiomics	ECG + radiomics
Women	F1_score		0.72	0.73	0.8	0.71	0.73	0.77
	Accuracy		0.75	0.75	0.8	0.73	0.75	0.78
	Sensitivity		0.67	0.67	0.79	0.68	0.68	0.76
	Specificity		0.83	0.83	0.81	0.78	0.82	0.8
	AUC		0.79 (± 0.09)	0.82 (± 0.03)	0.88 (± 0.07)	0.78 (± 0.13)	0.83 (± 0.04)	0.85 (± 0.05)
Men	F1_score		0.85	0.74	0.84	0.86	0.73	0.84
	Accuracy		0.85	0.75	0.84	0.86	0.74	0.84
	Sensitivity		0.79	0.7	0.81	0.82	0.69	0.81
	Specificity		0.92	0.8	0.87	0.9	0.79	0.87
	AUC		0.89 (± 0.05)	0.83 (± 0.06)	0.89 (± 0.06)	0.89 (± 0.04)	0.82 (± 0.06)	0.89 (± 0.05)

The standard deviation is indicated in parenthesis.

Finally, we extended the statistical analysis of phenotyping prevalent AF by selecting only the cases with patients of AF with a sinus rhythm without being differentiated with a normal ECG of a healthy patient and randomly matched with the healthy cohort with N = 45 and N = 49 for men and women, respectively. Again, the best added value of adding radiomics is for women reaching an 0.72 of AUC vs 0.54 (p < 0.05). The sensitivity increased significantly compared with ECG alone (0.65 vs 0.72). The best general model combining women and men was ECG + radiomics with an AUC of 0.61. For men the most predictive model was using radiomics with an 0.59 of AUC. Then, we observe that using radiomics in this scenario, the prediction improves for all cases, particularly in women. A summary of the results is shown in Table 8.

**Table 8:** Average performance of the models when the AF patients have a normal sinus rhythm and a normal ECG.

		ECG	Radiomics	ECG + radiomics
All	F_score	0.49	0.55	0.6
	Accuracy	0.53	0.53	0.6
	Sensitivity	0.51	0.57	0.62
	Specificity	0.56	0.49	0.57
	AUC	0.54 (± 0.11)	0.59 (± 0.12)	0.61 (± 0.08)
Women	F_score	0.60	0.6	0.68
	Accuracy	0.54	0.59	0.66
	Sensitivity	0.65	0.66	0.72
	Specificity	0.44	0.52	0.6
	AUC	0.54 (± 0.23)	0.67 (± 0.16)	0.72 (± 0.15)
Men	F_score	0.49	0.58	0.5
	Accuracy	0.49	0.58	0.54
	Sensitivity	0.49	0.57	0.49
	Specificity	0.51	0.6	0.59
	AUC	0.45 (± 0.14)	0.59 (± 0.19)	0.56 (± 0.19)

The standard deviation is indicated in parenthesis.

## Discussion

In this study we demonstrate the feasibility and clinical utility of using an integrative electro-anatomic model for AF diagnosis. We demonstrate the usefulness of these models in understanding phenotypic alterations that occur in AF. Importantly, we identified different electro-anatomical remodeling patterns in male and female patients with AF. Our findings indicate the usefulness of a more integrative approach to disease in women, who may have more subtle phenotypic alterations than men, particularly in the early disease stages.

As ECG is the main clinical tool for AF diagnosis, we expected ECG to have better results than radiomics alone, as was shown in the results for the men and general models. However, we found lower performance of the ECG model for women than men in AF. This behavior is clearly seen when the models are split into female and male subjects. The combination of ECG with radiomics predictors was able to improve the model performance among female subjects. Radiomics showed less added value for men, however the most relevant features selected by the Chi-Squared test were radiomics-based features, particularly from the left atrial. Although, it did not improve the model’s overall accuracy, this finding suggests that radiomics features may precede ECG changes in both men and women.

The underlying mechanisms of the sex differences in AF are incompletely understood. The main driving factors reported in the literature are higher body mass index, larger atria and ventricle size among males^36–38^. Notably, atrial enlargement has been linked to higher risk of incident AF^39^ and AF recurrence^40^. Moreover a study by van de Vegte et al. demonstrated that genetically susceptibility to AF increases indexed left atrial volumes and decreases LA ejection fraction^41^. On the other hand, these factors might also impact the interpretation of the ECG signal.

Our results suggest that women with AF have less overt ECG changes than men. Indeed, women have a higher heart rate at rest due to hormone effects, autonomic nervous system influences, and intrinsic properties of the sinus node. The P-wave is significantly shorter as well as the PR interval and the QRS duration. QT has also a more prolonged corrected interval in women^42^. As an example, prolonged QT interval possibly cause lower sensitivity for ECG in women with leading to false positive cases. Moreover, shorter P-waves with lower amplitude might make ECG recordings susceptible to noise and motion artifacts^43^. This means that the subtler radiomics feature changes are important for improving AF detection in women.

Due to the more pronounced ECG changes among male participants our model can differentiate between cases and controls with high accuracy using these features alone. Notably, radiomics features appear dominant in the combined models even for men. This suggests that radiomics features are more sensitive at picking up AF-related alterations and these changes may complement the information derived from the ECG.

We also performed an extension including only the patients with the diagnosed AF who were in sinus rhythm at time of their ECG. As expected, ECG was not able to distinguish between healthy and unhealthy participants. However, the inclusion of radiomics substantially improved the model performance, particularly in women. Importantly, increased atrial volume^44^ and atrial fibrosis^45^ might serve as a substrate for AF, and these alterations can be picked up by radiomics features. Although further information is needed to better describe the link between atrial radiomics features and biological precursors of AF.

The utility of artificial intelligence-based methods has been already demonstrated in the detection of AF, importantly sex differences are rarely addressed in these studies. The Apple Heart Study assessed the ability of an irregular pulse notification algorithm to identify AF in 419,297 (42% female) individuals^46^. Overall, 2161 (21% female) participants received a notification and 34% of cases were clinically confirmed from the total number of users detected by the smartwatch. In the study positive predictive value of an irregular pulse notification was 0.84 (95% CI, 0.76–0.92), supporting the ability of the algorithm to correctly identify atrial fibrillation, mainly among white male subjects. Notably, the datasets collected among smart device users rarely permit the assessment of sex differences, as man are more likely to own these devices in the first place^47^. AI applications are also used in the monitoring^48^, risk stratification^49^ and management of AF patients.

As a future work, we will extend this work to other cohorts to generalize the models and validate them to external data. With the inclusion of more data, we will also explore deep learning techniques combining all leads with the features that we identified in this work to improve the model accuracy. Moreover, we will also differentiate between certain types of atrial fibrillation to find phenotypes in each category instead of atrial fibrillation patients in general. We will also test the utility of the present model to predict incident AF.

### Limitations

Our ascertainment of AF status relied on clinical diagnoses. A limitation of this approach is that we would not capture as yet clinically unrecognized AF cases. As a result, some of the participants labelled as controls in our study may have low burden or paroxysmal AF that is not yet clinically identified. The impact of such misclassification would be attenuation rather than spurious high performance of our models. Additionally, the models were not validated externally limiting the generalizability of our results.

## Conclusions

In this study of the UK Biobank participants we demonstrated that an ECG-based model had lower accuracy to detect AF in female subjects compared to males. The inclusion of CMR radiomics combined with ECG increased the model performance in women. Especially CMR derived radiomics shape features of the LA had robust role in the betterment of our models, suggesting the critical role of atrial remodeling in the disease mechanism of AF. The main universal implication is that a combined approach of ECG and atrial imaging might lead to better assessment of female participants suspected of AF.

As a further layer of our analysis we selected prevalent AF patients with normal ECG tests, here, we found that all models got benefit from adding radiomics. But again, the clearest case was for women with the inclusion of radiomics with ECG features.

## Methods

### Population and setting

UK Biobank is a large-scale health database containing over a half million of participants aged between 40 and 69 years old and recruited across UK between 2006 and 2010. It is a powerful research resource including biomarkers, medical records, risk factors, clinical tests and physical measurements to study the most common and life-threatening diseases. The database is regularly updated with additional data, making it a potential source for research purposes. AF was detected through the Hospital Episode Statistics (HES) system, a database containing clinical details of all the admissions of the NHS hospitals in England, to provide a continuous follow-up of the participants.

### Study design and data

**Figure 4:**
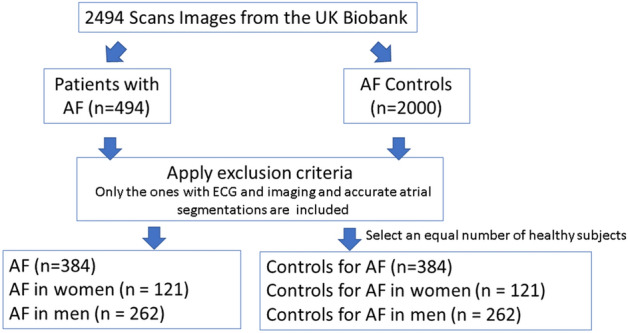
The process to select the data from the UK Biobank.

From the 495 prevalent AF cases in the UK Biobank cohort, we selected all the patients with AF who underwent both ECG and the CMR scan and the corresponding segmentation of the Left Ventricle (LV) and Right Ventricle (RV) cavities as well as the left and right atria were available (n = 383). To analyze the differences between sexes, we separated the data into female (n = 121) and male (n = 262) participants. Of these, 45 women and 49 men were in sinus rhythm at the time of their ECG recording. The healthy controls were defined as participants who were not diagnosed with AF and had a normal sinus rhythm on their ECG. For the healthy controls, we considered the first 2000 UK Biobank participants for computational purposes with ECG and CMR imaging. To avoid unbalanced models, the same number of healthy controls were randomly selected for each sex (Fig. 4).

### Feature extraction

The features of ECG were extracted in temporal, morphological and non-linear domains. Radiomics features were computed from the LV and RV segmentations in end-systole (ES) and end-diastole (ED) phases from short- and long-axis cine CMR images. The radiomics of the atrias were computed from the long-axis images. In this section, we will explain in detail how the features were extracted.

### Radiomics feature extraction

Radiomics features were extracted from the CMR images and the corresponding contours from three segmented ROIs: LV and RV cavities and LV myocardium in ED and ES in short axis. The segmentation of the ROIs was performed manually by expert cardiologists by defining the contours with points with a different label for each ROI using Cardiac MRI and CT Software (CVI42). The segmentations for each patient are exported in a single xml file containing the contour points for the RV, LV and MYO segmentations. In order to convert each contour into a binary mask, we developed an in-house software that transforms CVI42 contours into readable format contours^50^.

We also obtained the atrial segmentation using an automatic segmentation model based on a traditional U-Net architecture. First, a manual segmentation was performed by clinical experts in 764 datasets from Barts Heart Centre, UK^51^.Then, data augmentation techniques were used for generalizability of the model such as small rotations, random contrast adjustments and random intensity histogram shifting. The Adam optimizer was used with a learning rate of 0.0001 and 0.9 and 0.999 for first and second moments, respectively. The model was then trained with a batch size of 16 256 × 256 images with 100 epochs. The loss function used was cross entropy.

We computed the radiomics using the open-source python-based PyRadiomics library (version 2.2.0). To harmonize the images, the histogram matching technique was applied given a reference image. A binwidth of 25 was used to discretize the grey values of the image as it is the default parameter selected by pyradiomics. We extracted the relevant information present in the image by using three classes of features:

1. First Order Features: are histogram-based features related to the distribution of the gray level values in the tissue, without focusing on their spatial relationships.
2. Shape Features: describe geometrical properties of the organ, such as volume, diameter, minor/major axis and sphericity.
3. Texture Features: are derived from images and allow quantification of spatial relationships among pixels.

The shape radiomics of all the ROIs both for the short axis and long axis were all considered. However, the first-order and textural features were only considered from the LV myocardium as the other ROIs included parts such as the papillary muscles that can alter the intensity signals within the ventricular and atrial cavities. Shape features derived from the LV myocardium, LV, RV and LA, RA were selected for the analysis, while first-order and textural features derived only from the LV myocardium were used. A total of 420 atrial radiomics features were computed in long axis where each ROI contained the same number of features of each type (ROI shape n = 24, ROI first-order n = 36, ROI texture n = 150). Additionally, 262 radiomics features both for short and for long axis were included from each CMR study (LV shape n = 26, RV shape n = 26, MYO shape n = 26, LV myocardium first-order n = 36, LV myocardium texture n = 148).

### ECG feature extraction

We extracted the features of the ECG signals that are related according to literature with AF. We do not use the whole ECG signal as an input of the classification method to avoid overfitting. The ECG features for morphological, classical and non-linear features were computed using the open source code for ECG feature extraction in AF implemented in Matlab and mainly based on Physionet library^52^.

Firstly, classical ECG features were extracted based on morphological features in time domain including heartbeat intervals, analysis of QRS, QT, PR, R-R intervals and amplitude.

For robustness, morphological features in frequency domain were also extracted including power spectral density of the R-R intervals and frequency bands (ultra-low, very low, low and high frequency and Ratio of low- to high-frequency power).

Finally, non-linear features were also considered as the model of the heart is not only linear but also involves a nonlinear contribution. In this work, Poincaré Plot was used to extract non-linear features in ECG. Poincaré Plot is a 2D dimensional scatter plot where each point represents the RR interval as a function of the previous RR interval. The Poincaré analyzes quantitatively the shape of the plot which provides rich information of the behavior of the heart. For example, the plot for a patient with AF has a more circular shape than a healthy subject that is similar to a comet along the line of identity^53^.In order to determine the geometric appearance of the plot quantitively, some techniques such as ellipse fitting, correlation coefficient and histogram-based methods were implemented. Additionally, the Sample entropy was computed to measure the complexity of the time series^54^.

We proposed a multi-domain ECG feature extraction method including classical, non-linear and frequency domain features with a total number of 116 features. The second lead was used to extract the features as both old devices and the wearable devices are using a single lead. According to literature, the second lead provides the most valuable information^55,56^ including P, QRS and T waves. For that reason, it is the most used within the single-lead ECG works and the one with better results from 12-lead ECG recordings^57^.

### Feature selection

Chi-squared test is applied to the features, and selects metrics statistically significantly linked to the outcome.

The Chi-squared test can be defined as given the data of two variables, we can get observed count O and expected count E. Chi-Square measures how expected count E and observed count O deviate from each other. The formulation is as follows:

$${\chi }^2 = \sum \frac{(O_{i} - E_{i})^2}{E_{i}}$$

A small *p*-value of the test statistic indicates that the corresponding features is dependent on the outcome, and it is an important feature. The statistical test returns each feature's importance score using the -log of the p-value. A large score value indicates that the corresponding feature is important. In our approach, the number of features selected was 30 as the model stabilizes after 30 features.

### Statistical analysis

The experiments were conducted using the Matlab 2021b software. The correlation between ECG and radiomics was performed using Pearson’s correlation.

We used the fscchi2 function to apply the Chi-Squared test to select the most relevant features. A hierarchical model was built by combining radiomics with ECG to show the added value of incorporating radiomics features into the model for women, men and for both sexes. For comparison, we built the ECG and Radiomics-based models alone.

The models were trained with a Support Vector Machine (SVM) technique which has been widely used in cardiovascular risk predictions^58,59^ due to its numerous advantages such as computationally efficient and robustness for real-world applications as well as the ability to find non-linear relationships through the kernel trick.

The models were tested following a nested cross validation also known as double cross-validation, in order to minimize a biased evaluation of the accuracy of the model. Nested cross validation is widely employed in the machine learning field and was mainly developed to work with small datasets. Compared to standard cross validation techniques, nested cross validation can help in the reduction of overfitting and alleviate the limitation of optimistic biases, especially in relatively small samples. Varma and Simon et al., showed that nested cross validation methods provide an almost unbiased estimate of the true error compared to standard k-fold cross-validation particularly when used for both hyperparameter tuning and evaluation^60–62^. The method is divided into two loops: the inner loop is responsible for the selection of the best parameters, and the outer loop estimates the generalization accuracy^62^. This procedure splits the data into training and test folds k times in an outer loop. For each training fold, the hyperparameter optimization process is performed in an inner loop and returns the best parameters that minimize the error following the same procedure of partitioning and rotating the training fold into training and validation sets. Using this scheme, the test folds are never used to build the model, decreasing the possibility of overfitting. Notice that we have ten models trained with different partitions of the data not a single partition, making this procedure robust and reliable. Additionally, all the data has been used for testing making the performance measurements more reliable.

**Figure 5:**
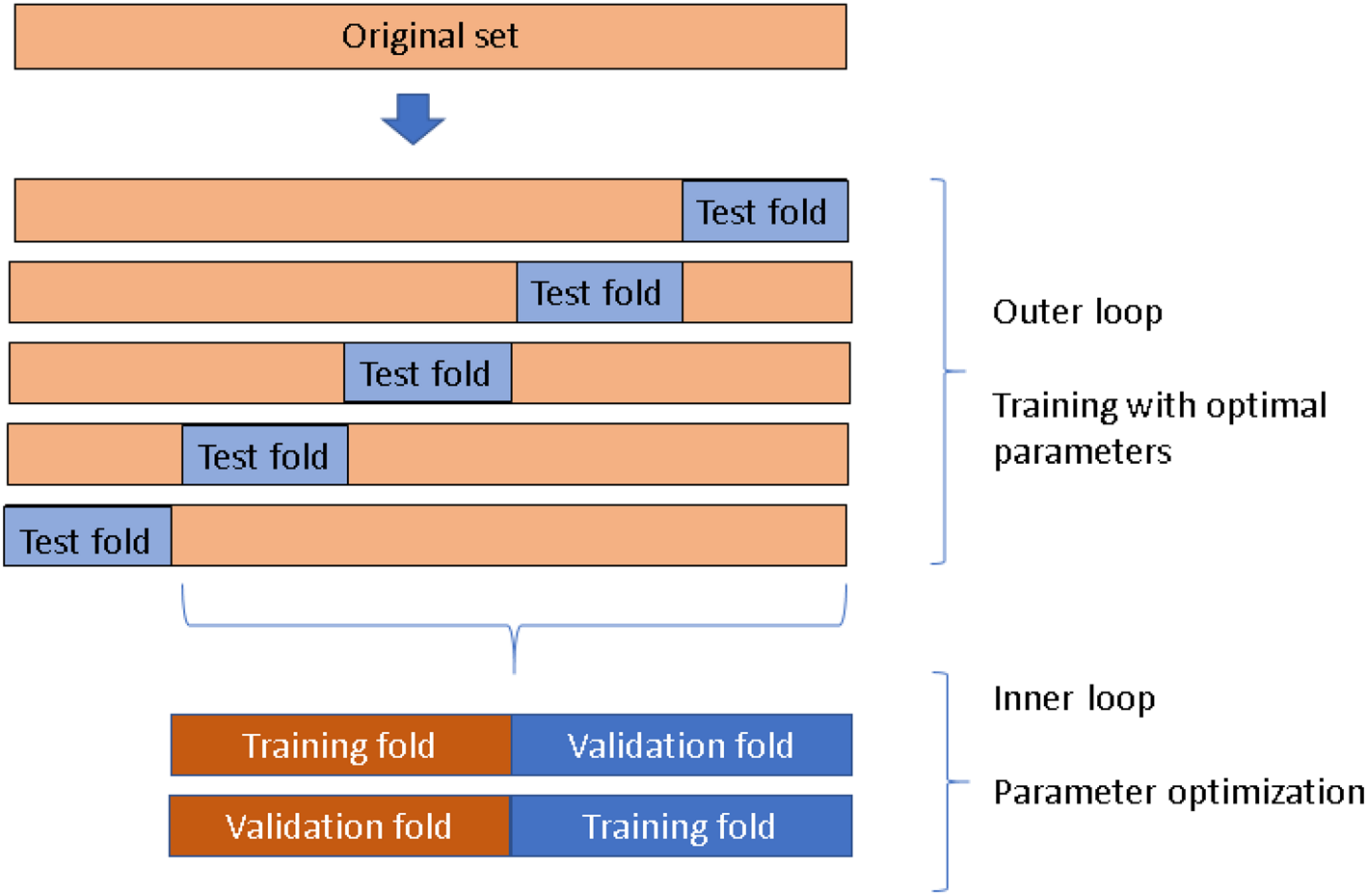
A nested cross validation scheme.

The hyperparameter optimization procedure was performed using greedy optimization which apply a brute force exhaustive search by trying each combination of each parameter. Five partitions are used for tuning the parameters of the SVM for each training fold in the inner loop (5-cross validation) and 10 cross-validation for the outer loop with partitions of 90% for training and 10% of testing in each outer fold. The summary of this procedure is shown in Fig. 5.

We computed to assess the performance of the models, the receiver operating characteristics (ROC) curve and area under the curve (AUC), as well as F1-score, accuracy, sensitivity and specificity over the test set. Additionally, Welch’s t-test was computed for group-wise comparisons. Several healthy partitions are randomly selected to show that the model does not depend on the selected data using different random seeds and we computed the ROC curve for each different partition of the healthy cohort.

To compare the models, a paired t-test on the distributions of AUC performances was performed to analyze the statistical significance in a nested cross validation framework^63^.

## Data Availability

The datasets generated and/or analysed during the current study are available online from the UK Biobank database, http://www.ukbiobank.ac.uk.
